# Instrumental Flirting: An Exploration of Charm in Decision-Making Groups

**DOI:** 10.3390/bs13070603

**Published:** 2023-07-20

**Authors:** David Henningsen, Mary Lynn Miller Henningsen

**Affiliations:** Department of Communication, Northern Illinois University, DeKalb, IL 60115, USA; henningsen@niu.edu

**Keywords:** flirting, flirting motivations, group decision making, group dynamics, instrumental flirting

## Abstract

The scholarship on flirting as a persuasive tactic in the workplace indicates that flirting can have negative consequences for task groups. The goal of this study was to extend the investigation of instrumental flirting by operationalizing this form of flirting as charm and by examining the consequences of charm in decision-making groups for the individual group members. In the current study, participants (60 women, 60 men) made decisions in four-person, mixed sex groups. The results of the study demonstrate that the use of charm was negatively associated with perceptions of group member task competence. Differences in perceptions of charm were also examined.

## 1. Introduction

Flirting is goal driven behaviors that promote ambiguity about the motivations of the flirter [1] Flirting behaviors can be courtship motivated. Courtship behaviors are behaviors that signal interest in a potential romantic or sexual relationship with the target of the flirting [2]. However, not all flirting is courtship motivated. Scheflen further identifies quasi-courtship behaviors as times when typical courtship initiation behaviors are employed for non-courtship purposes.

Investigating why individuals flirt, Henningsen [3] identified six flirting motivations: three courtship (i.e., sex, relational, and exploring) and three quasi-courtship (i.e., fun, esteem, and instrumental) (see also Frisby [4]). When individuals engage in flirting interactions with the goal of persuading another to assist them in accomplishing some non-courtship goal, it is referred to as *instrumental flirting*. 

In this study, we will look at instrumental flirting using Kray et al.’s [5] conceptualization of charm. Kray et al. introduced the construct *charm* to explore flirting as an influence tactic in one-on-one negotiations. Charm is defined as an impression management technique where the flirtatiousness of behaviors becomes more pertinent relative to the friendliness of the behavior. In other words, charm increases as behavior is viewed as more flirtatious than friendly. They found that women’s reported intent to use charm was associated with perceptions of their effectiveness during the negotiations (see study 1). 

Their findings provide some evidence that charm can be an effective tactic [5]. The use of flirting as an influence tactic, however, can have negative consequences in the workplace [6]. Henningsen and Henningsen found instrumental flirting in work groups was perceived as damaging to decision-making effectiveness. In the current study, we explore whether the perceived use of charm (i.e., using flirting as an influence tactic) in decision-making groups negatively affects co-workers’ perceptions of the *competence* (i.e., the use of logic and reasoning in decision-making) of fellow group members. 

### 1.1. Instrumental Flirting 

Instrumental flirting is a persuasive technique that employs quasi-courtship behaviors to influence a target to provide assistance or rewards [3]. The use of flirting to achieve instrumental goals has been studied in several contexts. For instance, Rowland et al. [7] reported that students perceive both men and women being able to influence their grades by flirting with faculty. Similarly, Gilbert et al. [8] found that flirting is encouraged as a component of service for waitstaff. 

Instrumental flirting also occurs in organizational settings. In a study of both workplace and social settings, Henningsen et al. [1] examined perceptions of instrumental flirting reported by a sample of working adults and students. Instrumental flirting was more common in the workplace than in the social setting.

An application of instrumental flirting can be found in Kray et al.’s [5] research on feminine charm (i.e., the use of charm by women). Kray et al. considered charm in multiple ways across four studies. They considered the planned use of charm, as well as the manipulations of flirtatious behaviors, using either transcripts (i.e., study 2) or instructions to negotiators (i.e., studies 3 and 4). Manipulating flirtatiousness produced mixed results. Promoting flirtatiousness using a transcript produced more favorable outcomes for the female negotiator. Interestingly, when female negotiators were instructed to employ more flirting behaviors in their interactions, women’s outcomes ranged from no difference (i.e., study 4) to worse (i.e., study 3).

Kray et al. [5] also operationalized feminine charm as other’s perceptions of the relative level of friendliness and flirtatiousness displayed in women’s behavior during a negotiation. The authors found that, as perceived flirtatiousness increased relative to friendliness, women performed better in one-on-one negotiations (i.e., studies 2 and 3). Furthermore, they found more mutual success for the negotiating dyad (i.e., study 4). These results demonstrate the potential benefit of charm as a persuasive tactic. 

While charm may be effective as a persuasive tactic in negotiations [5], it does have to be considered in the context of other work factors that are associated with the use of the tactic. As a specific tactic, charm has been investigated in a small body of research. Several lines of research, however, show that colleagues evaluate workplace romances quite critically. Workplace romance perceptions offer a framework for understanding possible implications of the use of charm. The use of charm parallels developing romantic relationships for instrumental purposes. Co-workers tend to negatively evaluate workplace relationships when the relationships are motivated by a desire for advancement (e.g., [9]). In a parallel line of research, Solomon and Williams [10] reported that using explicit social-sexual communication in the workplace is often evaluated negatively.

Additional research also recounts the hazards of workplace romance. For example, Chory and Gillen Hoke [11] examined workers’ perceptions of individuals involved in workplace romances. They found that when workplace romance was perceived as job motivated, the individuals in the relationship were less trusted by their co-workers (see also Horan and Chory [12]). Instrumental motivations associated with romantic or sexual relationships in the workplace have deleterious effects. 

In a study on feminine charm, Infanger et al. [13] examined perceptions of an adult sample recruited on Mechanical Turk. In an experimental design, participants read descriptions of women who described themselves as liking or disliking the use of feminine charm. They found that women described as enjoying the use of feminine charm were viewed as less likeable and more dominant than those who did not. 

Kray and Locke [5] explored the use of flirting in negotiations. Participants observed and rated half of a negotiation dyad. Target negotiators displayed either flirting (e.g., smiling and forward leaning) or neutral cues while utilizing the same scripts. In their study, the use of flirtation was evaluated as more manipulative than neutral cue use was. 

Instrumental flirting has been investigated in workplace decision-making groups using retrospective accounts. In an investigation of influence tactics, Henningsen and Henningsen [6] found that increasing levels of instrumental flirting as an influence tactic during decision-making negatively influenced perceptions of both decision quality and group cohesiveness. These findings indicate that negative impressions are downstream consequences of the use of instrumental flirting in the workplace. 

### 1.2. Biases in Perceptions of Charm in Groups

Flirting is inherently ambiguous [14]. The ambiguity is by design, as it allows for testing waters in a subtle manner that can easily be discounted if unwelcome. As a result, people do a poor job of identifying when flirting occurs [15]. In the context of group discussion, the combination of task demands with social demands are likely to complicate the perceptions of charm within discussion. These possible misperceptions mirror biases and misperceptions in allied literatures. 

Evidence about sexual misperception can be drawn from La France et al. [16]. Their meta-analytic review found sex differences for both male and female targets. Considering participants’ perceptions of face-to-face interactions, men perceived higher levels of sexuality than did women. This difference can be explained in a variety of ways. 

Harnish et al. [17] proposed a sexual bias explanation for sex differences in perceptions of cross-sex interactions (see also Abbey [18]). They argue that men view the world using a more sexualized lens than do women. When interpreting the same interaction, this explanation suggests that men will be more likely to attribute behaviors to sexual intent than women would. This has been described as a difference in baseline perceptions by Henningsen et al. [19]. In essence, it assumes men begin an interaction with higher baseline levels of sexual expectations than do women. The baseline difference reveals itself in higher overall perceptions of social-sexual behavior for men than women. Specifically, men should perceive more sexual intent than women in cross-sex interactions. 

Similarly, Shotland and Craig [20] proposed a difference based on perceptual thresholds. The threshold position rests on the idea of an initial threshold. When the threshold is reached, the behavior is evaluated as seductive rather than friendly. Based on this reasoning, men’s threshold is set lower than women’s, which results in men beginning to make attributions of sexual intent based on fewer cues. The difference in thresholds results in a sex difference in perceived sexual intent. This approach assumes that no differences emerge at levels below men’s threshold of social-sexual behavior.

Both Harnish et al. [17] and Shotland and Craig [20] found that men perceive more sexual intent in cross-sex interactions. Shotland and Craig further found, consistent with a threshold explanation, that men did distinguish between friendly and interested behavior. Other research indicates sex differences occur even at very low levels of perceived sexuality. For instance, Henningsen et al. [21] found that such sex differences occurred even when very few behaviors were identified as indicating sexual intent. This is consistent with a baseline approach and would indicate that if a threshold difference exists, the threshold is set very low for men. 

A theoretical position that is consistent with both the threshold and baseline explanations is error management theory (Haselton and Buss [22]). Rooted in evolutionary theory, error management theory proposes that people face the risk of making one of two types of errors when interpreting sexual intent during an interaction. A type I error occurs if behavior is interpreted as sexually motivated when that is not the sender’s intent. A type II error occurs if a person does not attribute a sexual motivation to behavior when that was the intent of the sender. According to the theory, evolutionary forces have caused men to bias their judgments in favor of type I errors. The theory holds that it is to men’s advantage to mistakenly perceive sexual intent from women’s behavior more so than to fail to recognize when that intent is present because of lost opportunities (i.e., the result of type II errors). The reasoning is derived from an evolutionary perspective that missed opportunities are more costly than rejected advances (i.e., the result of type I errors). Accordingly, the theory predicts that men will display a sexual over-perception bias when interpreting women’s behavior. 

In contrast, according to the error management theory, women focus more on questions of relational commitment in cross-sex interactions [22]. Because women face greater evolutionary risks from overestimating men’s potential relational commitment (i.e., type I error) than from underestimating it (i.e., type II error), women will display a commitment skepticism bias favoring a type II error. Henningsen and Henningsen [23] found support for both sexual over-perception and commitment skepticism in face-to-face, cross-sex interactions. Furthermore, women’s relational skepticism influenced their perceptions of men’s sexual intent, thus indicating a tendency toward sexual under-perception by women interpreting men’s behavior. 

Another possible explanation for misperceptions of sexual intent is offered by Koenig et al. [24]. The authors examined perceptions of sexual interest in cross-sex friendships. They proposed a projection model wherein the sexual interest of a cross-sex friend is predicted by the sexual interest of the person evaluating them. Overall, when controlling for the sex of the parties and the friends’ sexual interest, they found participants’ own level of sexual interest positively influenced their perceptions of their friends’ reciprocal interest. This effect mediated the relationship between sex and perceived sexual interest. Sex differences emerged in this case because men’s level of sexual interest has been found to be greater than women’s, and these differences drive the perceptions of sexual interest. Koenig et al. [24] also found evidence of men underestimating and women overestimating sexual interest in some cases. 

Hall et al. [15] examined perceptions of flirting in cross-sex interactions. Their findings were consistent with what they described as a base rate explanation. Drawing parallels from deception detection, they proposed that individuals may be biased toward assuming no flirting is occurring in interactions that are similar to truth bias in deception detection. Their findings were consistent with this assumption. They found that flirting detection accuracy was higher for non-flirting than for flirting interactions. In contrast with traditional sexual scripts, they did not find men flirted more than women. Furthermore, they did not find that men perceived higher levels of flirting from female targets than did women. This is inconsistent with error management theory. 

It is also possible that perceptions of sexual interest may vary due to differences in appropriateness judgements made by women and men [21]. Employing the logic of cognitive valence theory [25], Henningsen and Henningsen [21] argued that individuals apply cognitive schemas to understand social interactions. These schema influence how people respond to the interaction and how they interpret behaviors. For instance, individuals are less likely to view behaviors as sexually motivated when that is considered inappropriate. The authors found that women viewed sexual interest in cross-sex interactions to be less appropriate than did men. In addition, these appropriateness judgments negatively influenced both men’s perceptions of women’s sexual interest and women’s self-reported sexual interest. La France et al. [16] argue that this explanation may be most consistent with sex differences that emerge due to mode of presentation (i.e., observation of live interaction versus participation in live interaction) in perceptions of cross-sex interactions. 

### 1.3. Partner and Opponent Perceptions

The same behaviors that are used to flirt are also used to indicate affiliation or agreement. For example, Moore [26] categorized flirting behaviors used by individuals in a bar. Some of the behaviors identified as flirting (e.g., smiling, nodding, laughing) have alternatively been characterized as behaviors indicating agreement and affiliation [27]. As a result, observing the same behavior may result in attributions that the person is flirting or that they merely agree with the person.

In a decision-making group, group members may interact with others who agree with them as well as some who disagree. During group discussion, individuals would be expected to express agreement with other group members, particularly those arguing for the same position. Initial agreement is frequently based on the amount of information shared by group members prior to discussion [28]. Because of the similarity among flirting and agreement behaviors, more of these ambiguous behaviors (e.g., nodding and smiling) should be directed at partners than opponents. Ambiguous behaviors present the potential for over-attribution of charm, because behaviors that are not intended as flirting may be mis-categorized as flirting by others. 

### 1.4. The Current Study

In the current study, we focus on how the perceived use of charm as a form of instrumental flirting in decision-making groups influences group members’ perceptions of others’ task competence. The previous research on charm focused solely on women’s use of charm [5,13]. Related research indicates that charm is likely used by men and women. For example, both men and women report using instrumental flirting [3]. Men and women both reported instrumental flirting occurred in their work settings [1]. Judgements of men’s use of charm likely parallels women’s use of charm. We extend the research of Kray et al. [5] by considering perceptions of women’s and men’s use of charm. 

Henningsen and Henningsen [6] found that the use of instrumental flirting negatively influenced perceptions of group decision-making effectiveness, while the use of informational influence positively affected perceptions of effectiveness. Informational influence represents persuasion based on a desire to make the best possible decision [29]. It is typically associated with the use of logical arguments, facts, and evidence in group discussions [30]. We examined perceptions of competence during group discussion by focusing on perceptions of group members as logical and rational. We anticipated that the perceived use of charm would harm perceptions of group members’ competence.

Past research on differences in perceptions of sexual intent in interactions has focused on social, dyadic interactions (e.g., [31]). By examining decision-making groups, we extend past research by considering the task interactions in groups rather than one-on-one. Research finds that men overestimate and women underestimate the social-sexual intent of their partners in cross-sex dyads [16]. We predict that men will perceive greater use of charm than will women in decision-making groups. 

The use of decision-making groups further allows us to consider the potentially biasing effects of evaluating partners and opponents in interactions. Flirting cues are ambiguous and share behaviors in common with signaling agreement. The groups were composed so that each participant interacted with one opposite sex partner and a male and female opponent based on initial decision preferences supported by each group member’s information packet. It was anticipated that group members would perceive more flirting behaviors from partners than from opponents.

**Hypothesis 1.**  *Perceived charm will be negatively associated with perceptions of task competence*.

**Hypothesis 2.**  *Perceptions of women’s charm will be higher for ratings by male group members than by female group members*.

**Hypothesis 3.**  *Perceptions of men’s charm will be lower for ratings by female group members than by male group members*.

**Hypothesis 4.**  *Group members will report more charm from their partners than from their opposite sex opponents during group discussion*.

## 2. Method

### 2.1. Participants

Participants were recruited from upper division courses in an organizational communication emphasis at a large Midwestern university. Based on seminal studies, we relied upon in formulating our hypotheses [5,18,31], we targeted a sample size of 120 participants to meet or exceed the samples used in those studies. Because students participated in the study from the same classes, it is possible that some participants knew each other before participating. To offset this risk, we employed random assignment to groups. 

A student sample was employed due to the need to have participants work together in a controlled lab setting. Individuals (age: *M* = 21.48, *SD* = 1.10) were randomly assigned to four-person groups (*N* = 30). Each group included two male and two female group members. Approximately 17% reported being African American, 4% reported being Asian American, 71% reported being Caucasian American, and 2.5% reported being Latino/a American. The remainder selected other or did not report on ethnicity. This study was conducted with IRB approval. 

### 2.2. Procedures

Roughly 20 participants reported for each session. Each participant was randomly assigned to a group, and groups were spaced separately. Random assignment was used to promote zero-history groups. Each group consisted of four members: two women and two men. Information was distributed so that each group had two dyads consisting of one woman and one man. The first dyad received information favoring candidate A. The second dyad received information favoring candidate B. Members with the same information were partners in all analyses. Members who received different information were opponents for all analyses. Each group member had an opposite sex partner and a male and a female opponent based on information distribution. 

Group members were seated together in a classroom setting. After assignment to groups, the decision task was explained to participants. Group members were provided a written description of the criteria that they would use to make the decision and the information about the decision options. Information was distributed so that each group had two cross-sex dyads who should favor competing candidates. Before group discussion, each group member recorded their initial decision preference on individual forms.

After recording initial preferences to allow for a check of the partner manipulation, groups discussed the decision until a unanimous consensus was reached and recorded on a group decision form. Group decisions were also used as a manipulation check. Groups were given as much time as needed to reach consensus. Following the selection and recording of a preferred choice, each member filled out an individual questionnaire regarding the group discussion. Group members provided evaluations of themselves (i.e., self-ratings), of their partners (i.e., partner ratings), and of their female (i.e., female opponent) and male (i.e., male opponent) opponents. After completing the questionnaire, participants were debriefed and dismissed. 

### 2.3. Materials

Group members were assigned to select among three prospective student applicants for a position. A written set of decision-relevant criteria was provided on how the decision was to be made. Each criterion allowed for direct comparisons in which one of the candidates could be recognized as superior (e.g., higher undergraduate GPA). 

Group members received written descriptions of each candidate. Each group held 12 items of information about each of three candidates. Two of the candidates (i.e., superior candidates) scored the best for six of the criteria and were inferior to at least one other candidate in the other six criteria. The third candidate (i.e., inferior candidate) was equal to the best score in three of the categories and inferior to at least one other candidate in nine criteria. The information set was designed so that the two superior candidates were clearly preferable to the inferior candidate, but the superior candidates were comparable to each other. 

Information about the candidates was distributed among group members so that two cross-sex dyads favoring different candidates within each group were expected. Each dyad held information that initially favored one of the two superior candidates. We used the information distribution to create partner and opponent dyads within each group based on expected initial preferences. No member received information that contradicted information held by another group member, though competing dyads received different information to promote different preferences. Information was distributed so that each dyad held six items favoring their preferred candidate and three favoring the other superior alternative. 

### 2.4. Measures

#### 2.4.1. Charm

Our measures of women’s and men’s use of charm focused on perceptions of group member’s relative flirtatiousness and friendliness during a group interaction [5]. To assess perceptions of the use of charm, perceptions of group members’ flirtatiousness and friendliness were measured. Each group member was assigned an identifier (i.e., Group member A, B, C and D). Participants rated all four group members, including themselves, using those identifiers. Partners were always either members A and B or members C and D.

Flirtatiousness: Consistent with Kray et al.’s [5] characterization, flirtatiousness was designed to reflect flirtatious, sexual behavior. Items used to assess the perceptions of women’s and men’s flirtatious behavior during the negotiation were adapted from Abbey and Melby [31]. A three item, semantic differential measure was employed (i.e., Indicate your impression of this group member during the interaction: Sexy–Not sexy, Promiscuous–Not Promiscuous, Seductive–Not seductive). Each person rated the group members’ flirtatiousness, including a self-rating. Scores ranged from 1 to 6. Higher scores indicated more perceived flirtatiousness in the interaction. See Table 1 for means and standard deviations. 

Friendliness: We used a five item, semantic differential measure generated for this study to tap perceptions of group members’ friendliness during the negotiations (i.e., Indicate your impression of this group member during the interaction: Friendly–Unfriendly, Warm–Cold, Sociable–Unsociable, Likeable–Unlikeable, Kind–Unkind). These items were selected to reflect Kray et al.’s [5] characterization of friendliness as friendly and warm. Individuals rated themselves and their fellow group members on how friendly they were perceived to be during the interaction. Scores ranged from 1 to 6. Higher scores indicated more perceived friendliness during the interaction. See Table 1 for means and standard deviations.

Perceived charm: Kray et al. [5] operationalized charm as ratings of friendliness minus ratings of flirtatiousness. Using this method, greater perceptions of charm are represented by smaller numbers. Alternatively, we calculated charm by subtracting ratings of perceived friendliness from ratings of perceived flirtatiousness, (women: *M* = −2.08, *SD* = 1.62; men: *M* = −2.17, *SD* = 1.63). In this way, higher scores reflected higher levels of charm. The negative mean indicated that group members tended to perceive more friendliness than flirtatiousness during group discussion. 

#### 2.4.2. Task Competence

We created a four item, semantic differential measure to examine perceptions of group members’ competence (i.e., Indicate your impression of this group member during the interaction: Logical–Illogical, Smart–Not smart, Sensible–Not sensible, Rational–Irrational) following group interaction. Individuals rated their perceptions of the other group members during the interaction. Scores ranged from 1 to 6. Higher scores indicated more favorable ratings of competence. See Table 1 for means and standard deviations. 

## 3. Results

Correlations among the principle measures are presented in Table 2. Individuals were nested in groups in this experiment. For Hypothesis 1, we used participants as the level of analysis, because we were examining continuous variables using a test of association. This required us to account for group effects. Group effects occurred when membership in a group influenced group members’ behavior in a way that was unique to that group. Thus, individuals in group 1 may have behaved differently than those in group 2. We accounted for group effects by conducting generalized linear modelling in SPSS. Individuals were nested in groups in our design. Generalized linear modeling accounted for group effects to allow the effects of group membership to be distinguished from the effects of the predictor variables on the criterion variables. 

In Hypotheses 2 through 4, we used the group as the unit of analysis, because we were employing an ANOVA design to look at differences between means. Each score represented a group score rather than an individual score. For instance, when considering self-ratings of charm by women, the score was averaged across the two women who were members of the group and represented the self-reported charm for women in that group. In these analyses, degrees of freedom were based on the assumption that each unit was a group (i.e., *N* = 30) rather than each unit being an individual participant (i.e., *N* = 120). 

### 3.1. Manipulation Check

The experiment was designed so that mixed sex dyads would favor competing options in a group decision-making task. This created partner and opponent dyads based on the information held by members prior to discussion. We examined whether the information manipulation was successful by examining whether the group members initially selected the option favored by the information they were provided. For the first set of information, 45 of 60 participants selected the option favored by their information. The favored option was significantly more likely to be selected than the alternative, with a χ^2^ = 15.00 and a *p* < 0.05 indicating the manipulation was successful. In the second information set, 51 of 60 participants chose the option favored by their information. The favored option was significantly more likely to be chosen than the alternative, with a χ^2^ = 29.40 and a *p* < 0.05, again indicating the success of the manipulation. The results of the manipulation check indicated that the information distribution did support cross-sex partnerships and competing positions.

The ultimate group choice between the two favored options indicated no significant difference for the group preferences, with a χ^2^ = 0.13 and a *p* > 0.05. Sixteen groups selected the option favored by the first dyad, while 14 selected the option favored by the second. This indicates that the information held by the groups was balanced with regard to the top two options. Thus, the information distribution did not bias group decisions. Neither set of information appeared more persuasive than the other. 

### 3.2. Tests of Hypotheses

#### 3.2.1. Charm and Perceptions of Task Competence

We tested Hypothesis 1 by utilizing generalized linear modeling. In order to test Hypothesis 1, we performed separate analyses for each potential rater for male and female targets. We explored the impact of perceptions of charm on perceived competence as rated by individuals’ partners, their male opponents, and their female opponents. For all tests, the competence of the group member was evaluated as the criterion variable controlling for the effects of group membership and assessing the effect of charm.

We first examined the perceptions of women’s charm as rated by the women’s partners, female opponents, and male opponents. The omnibus tests comparing the proposed models to the intercept-only models were significant for partner, χ^2^ = 98.34 and *p* < 0.05, female opponent, χ^2^ = 65.78 and *p* < 0.05, and male opponent, χ^2^ = 69.90 and *p* < 0.05,. Charm produced a significant negative association with perceptions of women’s competence for ratings by partners and by female opponents but not for male opponents (see Table 3), which partially supported Hypothesis 1.

We also examined the perceptions of men’s charm as rated by the men’s partners, female opponents, and male opponents. The omnibus test comparing the proposed models to the intercept-only models were significant for partner, χ^2^ = 37.79 and *p* < 0.05, female opponent, χ^2^ = 63.18 and *p* < 0.05, and male opponent, χ^2^ = 55.68 and *p* < 0.05. Charm produced a significant negative association with perceptions of partners and female opponents though not for male opponents (see Table 3). Once again, partial support emerged for Hypothesis 1.

#### 3.2.2. Perceptions of Use of Charm

Hypotheses 2 through 4 were tested using a 2 (Sex of target: male or female) × 2 (Sex of rater: male or female) × 2 (Group dyad: Self, partner or male opponent, female opponent) within group ANOVA design with the group as the unit of analysis. Significant main effects emerged for the sex of the rater—*F* (1, 29) = 17.71, *p* < 0.05, *partial* η^2^ = 0.39, group dyad, *F* (1, 29) = 9.39, *p* < 0.05, *partial* η^2^ = 0.25—and sex of target—*F* (1, 29) = 4.42, *p* < 0.05, *partial* η^2^ = 0.35. In addition, there was a significant interaction between sex of target and the group dyad—*F* (1, 29) = 11.68, *p* < 0.05, *partial* η^2^ = 0.29. No other effects were significant (see Table 4 for means and standard deviations).

The sex of the rater main effect indicated that men perceived significantly higher levels of charm than did women. Upon examining the means for the perceptions of women’s use of charm (see Table 4), we find both male partners and male opponents viewed more charm than was reported by female opponents and women’s self-reports. Thus, Hypothesis 2 is supported. 

The means for the perceptions of men’s use of charm presented a different picture. Although men self-reported significantly higher levels of charm than either their female partners or opponents perceived, male opponents did not follow the hypothesized pattern. For men, male opponents’ ratings did not differ significantly from those of the female opponents or partners (see Table 4). The results partially support Hypothesis 3.

The significant main effect for group dyad is consistent with Hypothesis 4. However, contrast tests revealed that this effect was primarily driven by differences between self-ratings and those of opponents. Significant differences did not emerge between partner ratings and opponent ratings for men or women (see Table 4). Hypothesis 4 is not supported. 

The significant main effect for sex of target indicates that group members reported higher levels of women’s charm than men’s charm during the interaction. However, the significant interaction between sex of target and the group dyad revealed a magic cell. Male and female opponents’ ratings of men’s use of charm, *M* = −2.10, SE = 0.16, reported less perceived charm than self and partners’ ratings of men’s use of charm, *M* = −1.55, SE = 0.15, male and female opponents’ ratings of women’s use of charm, *M* = −1.55, SE = 0.15, and self and partners’ ratings of women’s use of charm, *M* = −1.54, SE = 0.16. 

## 4. Discussion

Past research has found that social-sexual behaviors such as flirting [6] and workplace romance [32] can have negative consequences in organizations. However, prior research has not examined the occurrence of social-sexual behaviors in live, task related interactions. Our findings extend past previous research by showing their applicability in decision-making discussions. We found that charm has a significant, negative impact on perceptions of group members’ task competence.

We also extended the research on biases in perceptions of social-sexual behavior. Although past studies have examined biases in perceptions of both participants and observers [16], to date, no studies have examined perceptions in interactions involving more than two people. Our findings showed that biases influence perceptions of charm in interacting task groups. Specifically, we found that women’s use of charm tended to be overestimated by men, while men’s use of charm tended to be underestimated by both women and other men. Coupled with our findings concerning task competence, these biased perceptions are problematic for organizations. Finally, we extended Kray et al.’s [5] research in the current study by examining men’s charm in addition to women’s charm.

In the following sections, we consider how the results relate to our hypotheses. We consider the limitations of this study and directions for future research. Finally, we discuss the implications of this study. 

### 4.1. Charm and Competence Perceptions

Our results provide some support for Hypothesis 1. We predicted that the perceived use of charm by women during group discussion would be negatively associated with perceptions of task competence. For male partners and for female opponents, perceptions of women’s charm were significantly and negatively associated with their perceived task competence. However, although a negative association was also identified for male opponents’ perceptions of women’s charm, this effect was not significant. Our results for men’s charm paralleled those for women’s charm. We found that female partners and opponents associated men’s use of charm with lower levels of task competence, but male opponents once again did not significantly associate the use of charm with competence.

The effect for male opponents is consistent with Henningsen and Henningsen’s [21] application of cognitive valence theory. In that study, women were more likely than men to perceive the use of social-sexual behavior as inappropriate. Appropriateness judgement, furthermore, influenced the perceptions of social-sexual behavior. It is possible that the negative perception linked to the use of charm in this study (i.e., that it reduces perceived task competence) can be attributed to perceptions of the appropriateness of the behavior. If so, it would be expected that male opponents would display less negative reaction. 

### 4.2. Sex Differences in Perceptions of Charm

Given the downside of employing charm, misperception represents a risk in the workplace. We posit that the most accurate assessment of the use of charm are self-reports. Based on our definition that flirting is goal-motivated behavior, we accept that the sender determines how much flirting is occurring in an interaction. In Hypothesis 2, we predicted that men (i.e., male partners and opponents) would report higher levels of women’s charm than women (i.e., female opponents and self-reports) would. Our results supported the hypothesis. Women’s ratings of their own charm and women’s ratings of female opponents’ charm did not differ. Similarly, male partners’ and opponents’ ratings did not differ. Men did, however, report higher levels of women’s use of charm during the group decision-making task than women did. 

It is worth noting that both men and women tended to agree that women’s behavior in the interaction was better characterized by friendliness than by flirtation, with women rating friendliness almost two points higher on a six-point scale. Thus, differences were emerging at low levels of perceived charm. 

In Hypothesis 3, we predicted that men’s self-rated charm, as well as male opponents’ ratings of that charm, would be higher than female opponents’ or partners’ ratings. This hypothesis received only partial support. Although women’s (i.e., partners and female opponents) ratings were significantly lower than men’s self-reports, no difference emerged between male opponents’ and female opponents’ and partners’ ratings of men’s charm.

It is possible our findings for charm reflect the differences in base rate accuracy identified by Hall et al. [15]. Men self-report significantly more charm than women do. Thus, female opponents more accurately identified women’s relatively low use of charm, while male opponents underestimated men’s relatively greater use of charm. 

### 4.3. Partner’s and Opponent’s Views of Charm

We also explored whether shared positions in decision-making groups influenced perceptions of charm. We posited that behaviors that signaled agreement could be easily interpreted as flirting cues, thereby leading partners to attribute more behaviors to charm than opponents. 

Counter to our hypothesis, no difference emerged for perceptions of men’s and women’s charm based on their role within the group (i.e., partner or opponent). Our findings indicate that sharing the same initial position did not predict perceptions of charm. 

### 4.4. Limitations and Directions for Future Research

We examined perceptions of charm in the discussions of task groups. This represents a shift from past research on social-sexual workplace behavior, which has relied on recall [1] or scenario descriptions [12] to study behavior. A trade-off occurred because we relied on a student rather than a working sample and utilized zero-history groups. Although we believe we increased the internal validity by having participants evaluate a live interaction immediately after participating in it, we acknowledge the trade-off with external validity. Future research could address this concern by examining live interactions with a working sample of intact work groups. 

In addition, our measures relied on the perceptions of the participants. We did not record the flirting behaviors of group members. While the perceptions of charm are obviously important, examining behaviors associated with charm used in group discussion could provide a richer picture of the cues associated with differences in perceptions.

An additional limitation arises, because we did not record the sexual orientation or relational status of participants. Past research indicates sexual orientation [32] and relational status [33] can influence perceptions of social-sexual behavior in working settings. Future research could examine whether sexual orientation or relational status influence biases in perceiving charm as well as the how such perceptions relate to judgments of competence. 

We also used global measures of perceived flirtatiousness and friendliness. As noted, more targeted measures looking at perceptions of charm directed at the rater or another target could provide information about why differences emerge for the association between charm and task competence. In addition, the motivation attributed to social-sexual behavior influences how those behaviors are perceived [34]. Examining perceived motives for the use of charm could be enlightening. 

Finally, we posited that cognitive valence theory [25] could explain why male opponents did not behave as hypothesized with regard to perceptions of charm and competence. This explanation could be explored by employing a personality trait such as Machiavellianism, which should relate to appropriateness judgments. Other variables could also be examined to tease out differences among theoretical explanations. We provide no direct test of the different theoretical mechanisms used to explain perceptions of social-sexual behavior. In the future, critical tests could provide useful insights for businesses and organizations. 

## 5. Implications

Charm has been demonstrated to be an effective negotiation strategy, at least for women [5]. However, Kray et al. did not consider what trade-offs occurred when employing this strategy. We found that perceptions of charm were negatively associated with perceptions of task competence for group members other than male opponents. This indicates that individuals need to consider the consequences of choosing to use charm. 

Our findings have implications beyond the choice of using charm. Prior research has found differences in perceptions of social-sexual behavior in face to face, as well as cross-sex interactions [16]. In the current study, we extended those findings to both task and group settings. We found that misperceptions, defined as differences from individual self-reports of behavior, occurred for both men and women. However, whereas women’s charm was overestimated by men relative to women’s self-reports, men’s charm, as assessed by men’s self-reports, tended to be underestimated by all other parties. Given our findings concerning the perceptions of competence, this implies the strategic use of charm may have more risks for women than for men. Specifically, it may indicate perceptions of women’s competence in group tasks may be underestimated due to over-perception of their use of charm. 

## 6. Conclusions

Our findings offer a clear warning about the use of charm as an influence tactic in the workplace. Although past research has shown that charm can be an influential tactic [5], our findings reveal that success comes at a cost. On a group decision-making task, the perceived use of charm was negatively associated with perceptions of task competence by many other group members. 

The findings indicate an additional potential problem for organizations regarding the perceived use of charm. Charm tended to be misperceived in task groups. This occurred because women experience over-perception, while men experience under-perception of charm relative to their self-reported behaviors. If charm is, in fact, associated with influence this could mean that it is a more effective tactic for women than for men. However, it also means perceptions of women’s task competence are more likely to be penalized. 

Obviously, individuals need to be aware of how their behavior can influence the perceptions of others. However, our findings indicate it is also important for individuals in the workplace to carefully scrutinize their own perceptions. Misperceived behaviors can lead to biased impressions of co-workers. Research has shown that social-sexual behavior can influence career influencing decisions [35]. Our findings indicate these decisions may be influenced by biased judgments. Organizational effectiveness necessitates that individuals consider how biased perceptions may be affecting their judgments.

## Figures and Tables

**Table 1:** Measurement.

	α	*M*	*SD*
Competence			
Self	0.71	4.89	0.74
Partner	0.66	4.82	0.74
Male Opponent	0.69	4.82	0.79
Female Opponent	0.70	4.72	0.72
Flirtatiousness			
Self	0.75	3.58	0.87
Partner	0.77	3.16	0.84
Male Opponent	0.79	2.71	0.80
Female Opponent	0.81	3.18	0.85
Friendliness			
Self	0.72	5.06	0.60
Partner	0.74	4.78	0.67
Male Opponent	0.69	4.82	0.68
Female Opponent	0.72	4.77	0.70

**Table 2:** Correlations among variables.

	1	2	3	4	5	6	7	8	9
Sex of rater (1)	1.00								
Partner charm (2)	−0.13	1.00							
Partner comp. (3)	−0.06	−0.46 **	1.00						
Female opp. charm (4)	−0.23 *	0.74 **	−0.34 **	1.00					
Female opp. comp. (5)	−0.04	−0.41 **	0.73 **	−0.46 **	1.00				
Male opp. charm (6)	0.12	0.57 **	−0.30 **	0.69 **	−0.29 *	1.00			
Male opp. comp. (7)	−0.02	−0.42 **	0.67 **	−0.44 **	0.77 **	−0.37 **	1.00		
Self charm (8)	−0.15	0.69 **	−0.32 **	0.59 **	−0.32 **	0.44 **	−0.39 **	1.00	
Self comp. (9)	0.01	−0.44 **	0.78 **	−0.29 *	0.69 *	−0.27 *	0.73 **	−0.37 **	1.00

* *p* < 0.01; ** *p* < 0.001. For sex of rater, men are 0 and woman are 1.

**Table 3:** Effect of perceived charm on perceived female competence.

	B	SE	Lower Bound	Upper Bound	*p*
Female target					
Partner	−0.21	0.06	−0.33	−0.09	<0.01
Male opponent	−0.13	0.10	−0.31	0.06	0.19
Female opponent	−0.26	0.07	−0.39	−0.13	<0.01
Male target					
Partner	−0.16	0.07	−0.30	−0.02	0.02
Male opponent	0.05	0.06	−0.08	0.18	0.43
Female opponent	−0.40	0.06	−0.53	−0.27	<0.01

*N* = 120.

**Table 4:** Mean and standard deviations for women’s and men’s use of charm.

	M	SD
Women’s charm		
Self	−1.73 _ab_	1.10
Partner	−1.35 _cdef_	0.99
Opp. man	−1.24 _aghij_	0.88
Opp. woman	−1.87 _cgk_	0.99
Men’s charm		
Self	−1.22 _bklmn_	1.10
Partner	−1.89 _dhl_	1.01
Opp. man	−1.98 _eim_	1.07
Opp. woman	−2.21 _afjn_	0.96

*N* = 30. Scores calculated by subtracting perceived friendliness from perceived flirtatiousness. Higher scores indicate more charm. Scores with the same subscript differ significantly. Self-ratings reflect an individual’s rating of their own charm. Partner ratings are ratings of that person’s charm by the person’s partner. Opponent ratings are evaluations of that person’s charm by their male or female opponent.

## Data Availability

This data is not publically available.
